# Supplementary material for: Mechanism of Arp2/3 complex branch disassembly by human Coro7
Source: Nat Commun. 2025 Nov 6;16:9809. doi: 10.1038/s41467-025-64787-z (PMC12592476; doi:10.1038/s41467-025-64787-z)
Supplement: Supplementary file 1 — Supplementary Information [file 41467_2025_64787_MOESM1_ESM.pdf]

Supplementary Information for

Mechanism of Arp2/3 Complex Branch Disassembly by Human Coro7

Nooshin Shatery Nejad, Malgorzata Boczkowska, Rouba Hilal, Fred E. Fregoso, Kyle R. Barrie, Grzegorz Rebowski, Andrew J. Saks, Alexis M. Gautreau, Enrique M. De La Cruz, Roberto Dominguez

Correspondence: Roberto Dominguez Email: droberto@pennmedicine.upenn.edu

The supplementary information includes:

Supplementary Fig. 1 | Western blot analysis of crosslinking reactions
Supplementary Fig. 2 | Cryo-EM data processing workflow
Supplementary Fig. 3 | Cryo-EM map validation
Supplementary Fig. 4 | Binding of Coro7 constructs to F-actin in the ADP and ADP-BeF$_3$ states
Supplementary Fig. 5 | Force-dependence of branch lifetimes with and without Coro7 FL
Supplementary Fig. 6 | Electroporation of purified Coro7 proteins into Coro7 KO cells
Supplementary Fig. 7 | Loss of Coro7 increases perinuclear branched actin networks that colocalize with GBF1 at the ERGIC
Supplementary Fig. 8 | Evidence supporting the proposed Coro7 debranching model
Supplementary Table 1 | Primers and antibodies used in this study
Supplementary References

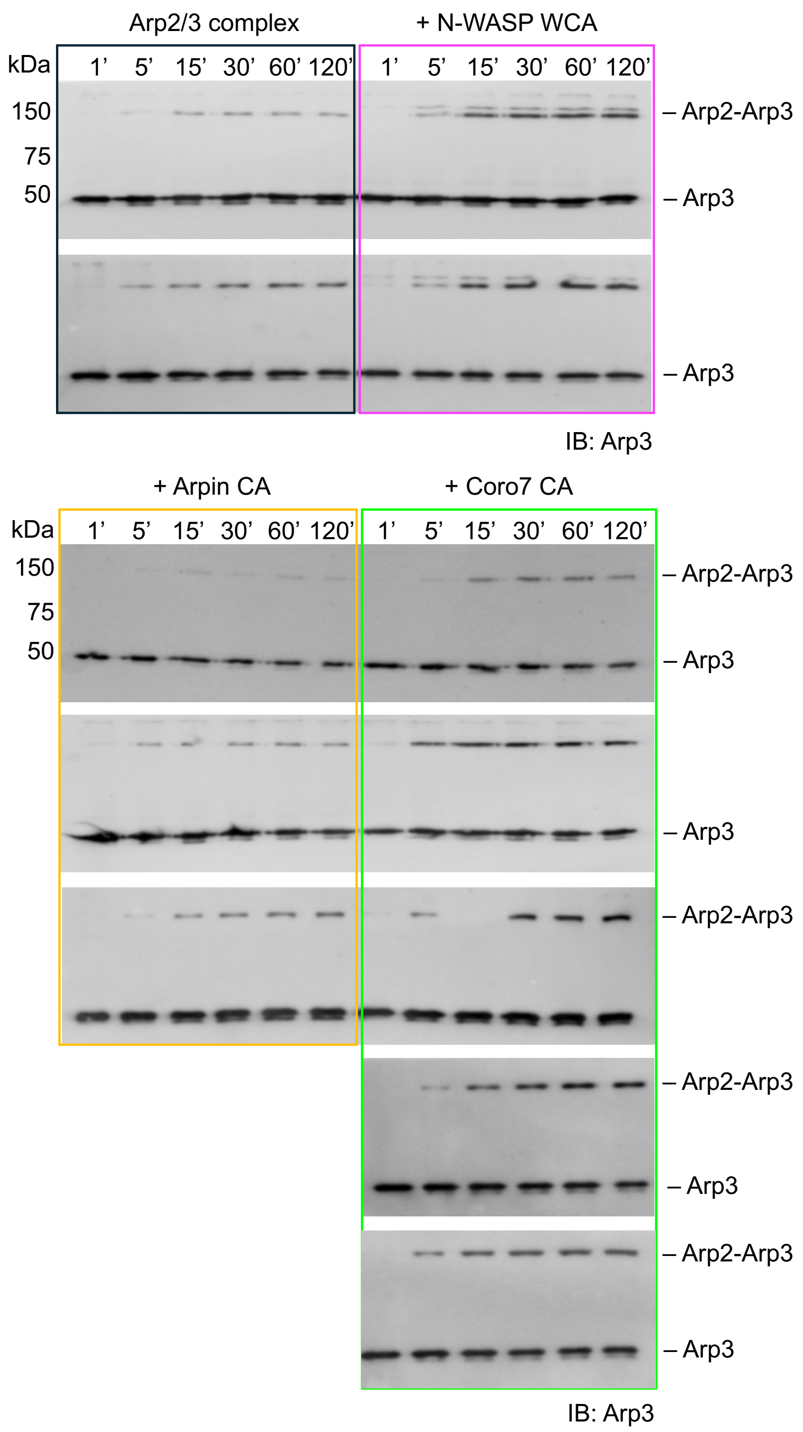

Supplementary Fig. 1 | Western blot analysis of crosslinking reactions. Western blot analysis of the crosslinked fraction of Arp2/3 complex alone or in the presence of N-WASP WCA, Arpin CA, or Coro7 CA, corresponding to the gels outlined in black, magenta, orange, and green, respectively. An anti-Arp3 antibody was used to detect the crosslinked Arp2–Arp3 band, which forms only when the Arp2/3 complex adopts the short-pitch conformation. Additional gels and densitometric quantification are shown in Fig. 2b of the main text. Source data are provided in the Source Data file.

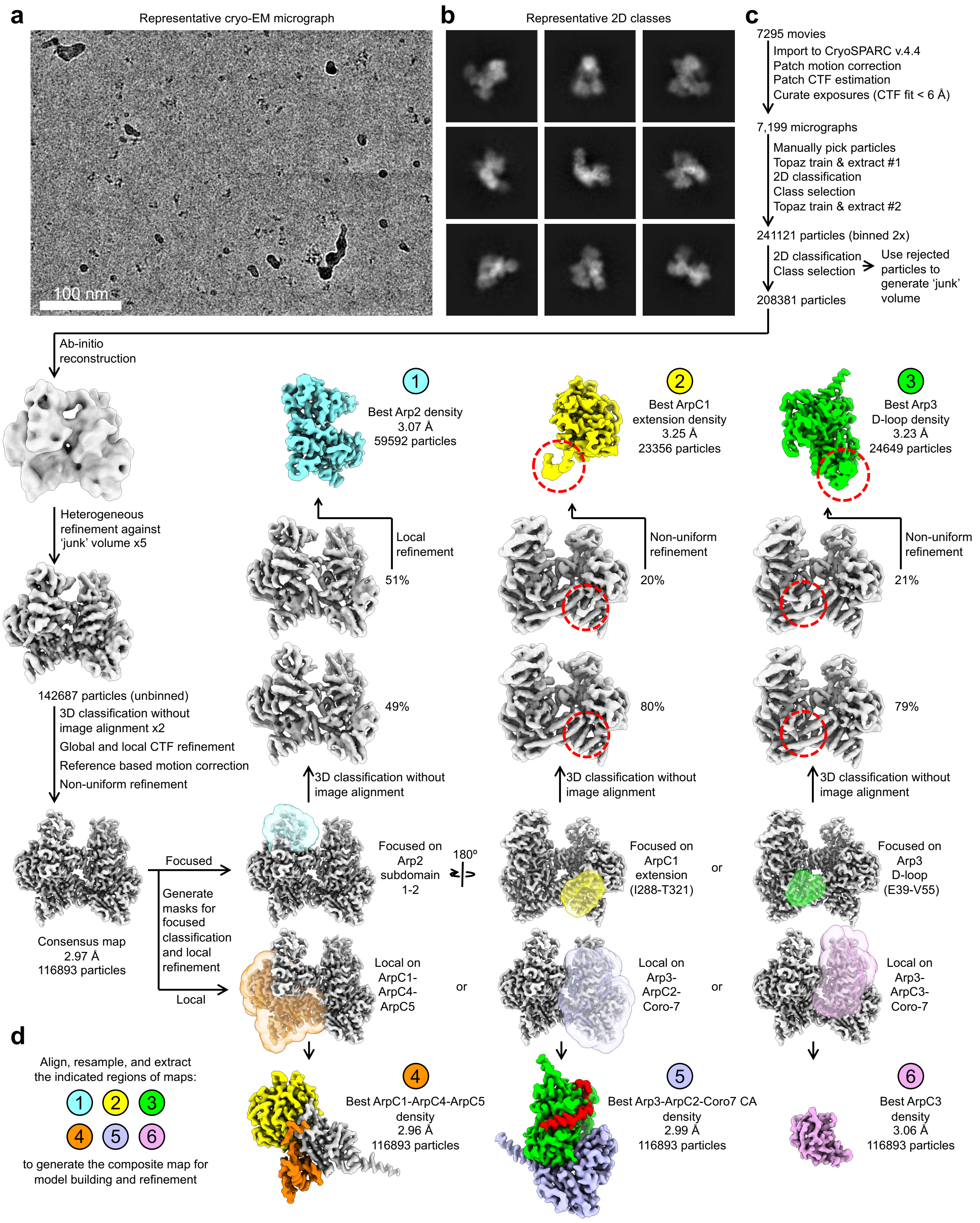

Supplementary Fig. 2 | Cryo-EM data processing workflow. a Representative micrograph (from a total of 7,295). **b** Representative 2D class averages. **c** Cryo-EM data processing workflow (see Methods for details). **d** Strategy used to generate the composite map for model building and refinement by combining the best-resolved regions from six maps, obtained through local masking and refinement.

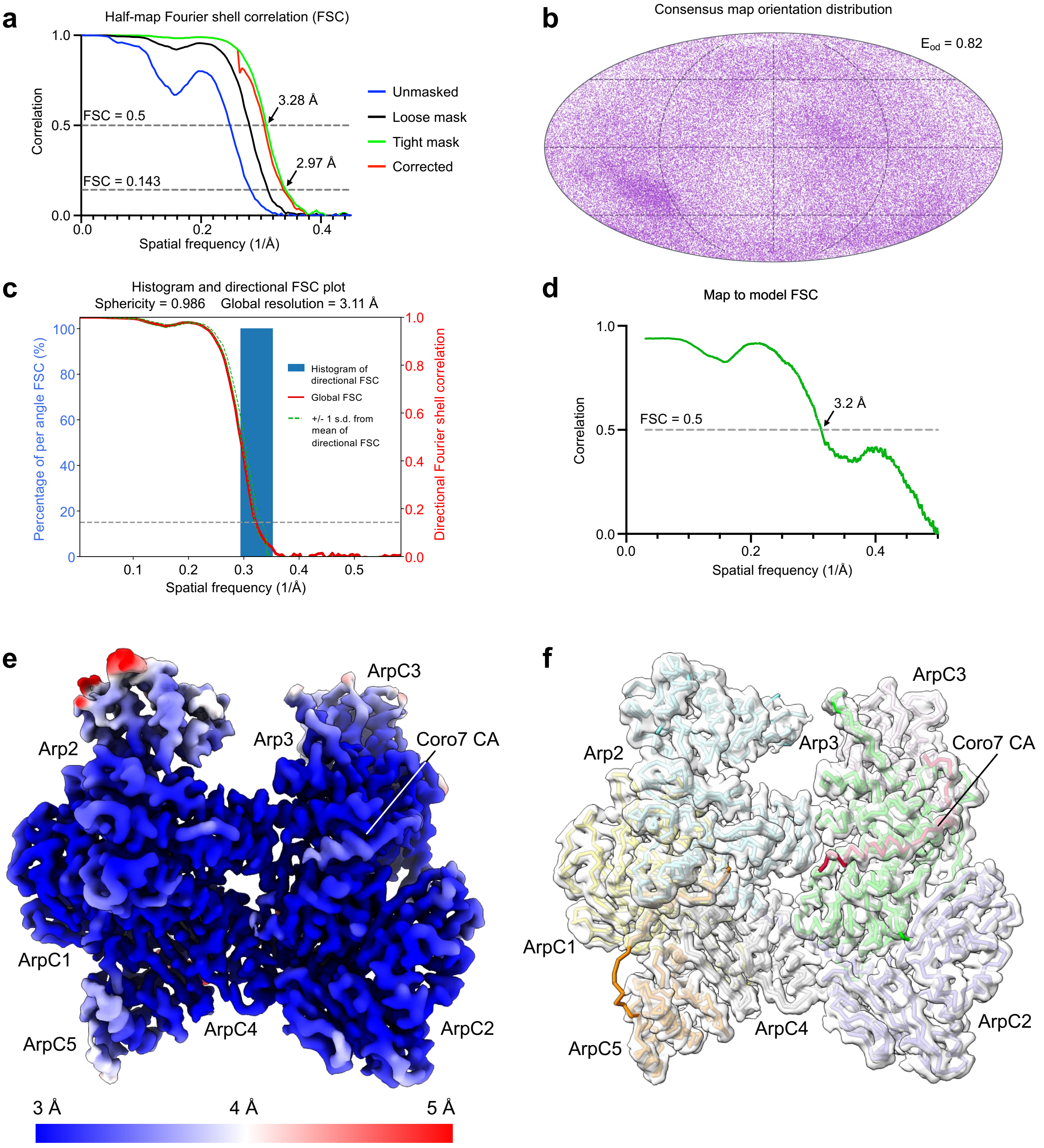

Supplementary Fig. 3 | Cryo-EM map validation. a Half-map Fourier shell correlation (FSC) resolution analysis of the consensus map (2.97 Å at FSC = 0.143). **b** Orientation distribution of particles used in the consensus map determined with the program cryoEF [1]. The consensus map has a calculated efficiency (E_{od}) of 0.82. **c** 3D Fourier shell correlation calculated using 3DFSC [2]. The consensus map has a sphericity of 0.986 and a global resolution of 3.11 Å. **d** Map to model FSC determined with the program Phenix [3]. **e** Cryo-EM map of Coro7 CA bound to Arp2/3 complex, colored by local resolution as indicated by the scale bar on the right. **f** Fit of Cα-trace of the structure to the cryo-EM map.

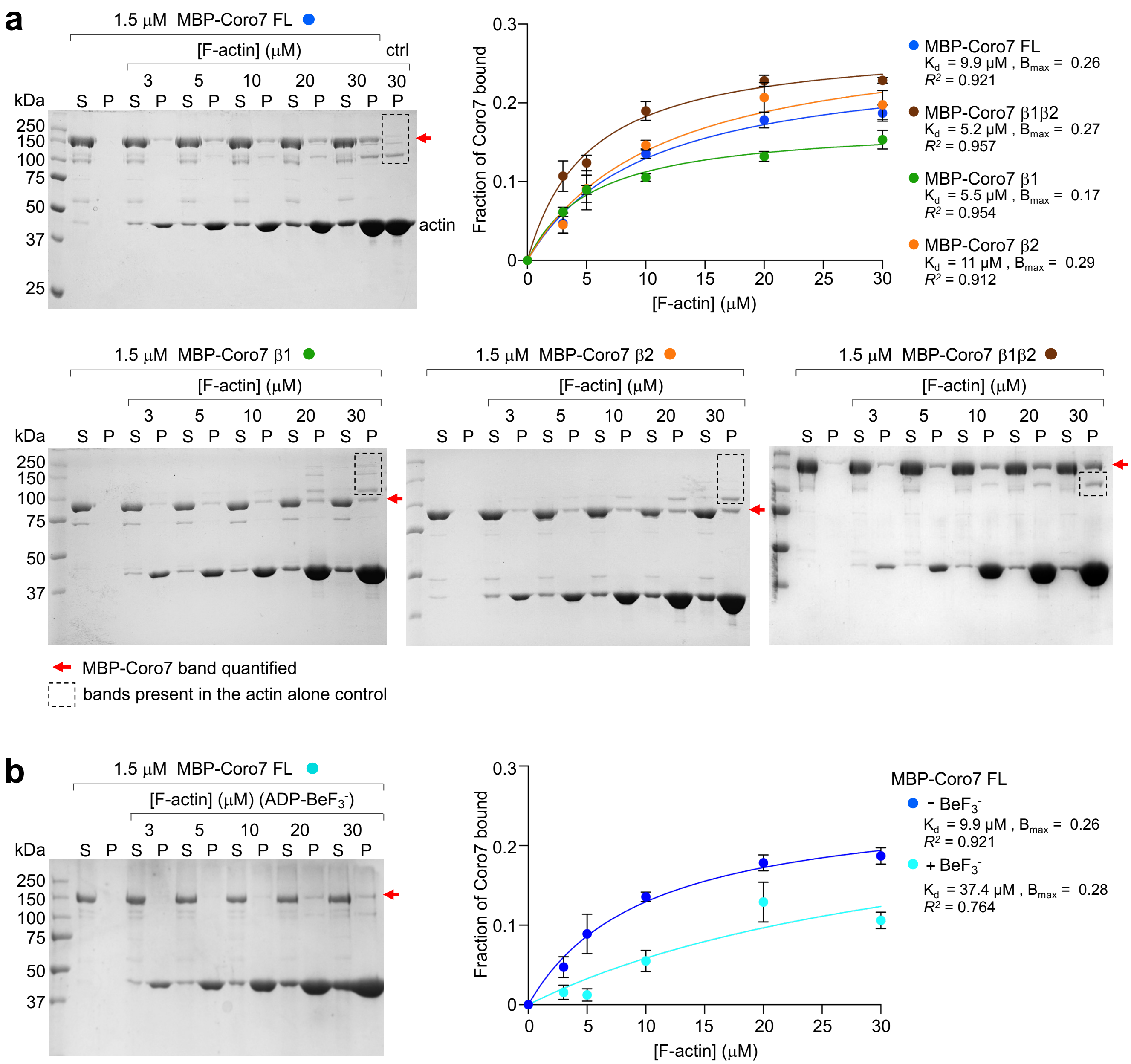

Supplementary Fig. 4 | Binding of Coro7 constructs to F-actin in the ADP and ADP-BeF₃ states. a Quantification of the fractions of MBP-Coro7 constructs FL, β1, β2, and β1β2 that cosediment with varying concentrations of ADP-F-actin, based on SDS–PAGE and densitometric analysis from three independent experiments. A representative gel is shown for each construct, with all gels provided in the Source Data file. **b** Quantification of the fraction of MBP-Coro7 FL that cosediments with varying concentrations of ADP-BeF_3^--F-actin compared to ADP-F-actin, based on SDS–PAGE and densitometric analysis from three independent experiments. A representative gel is shown for each experiment, with all gels provided in the Source Data file.

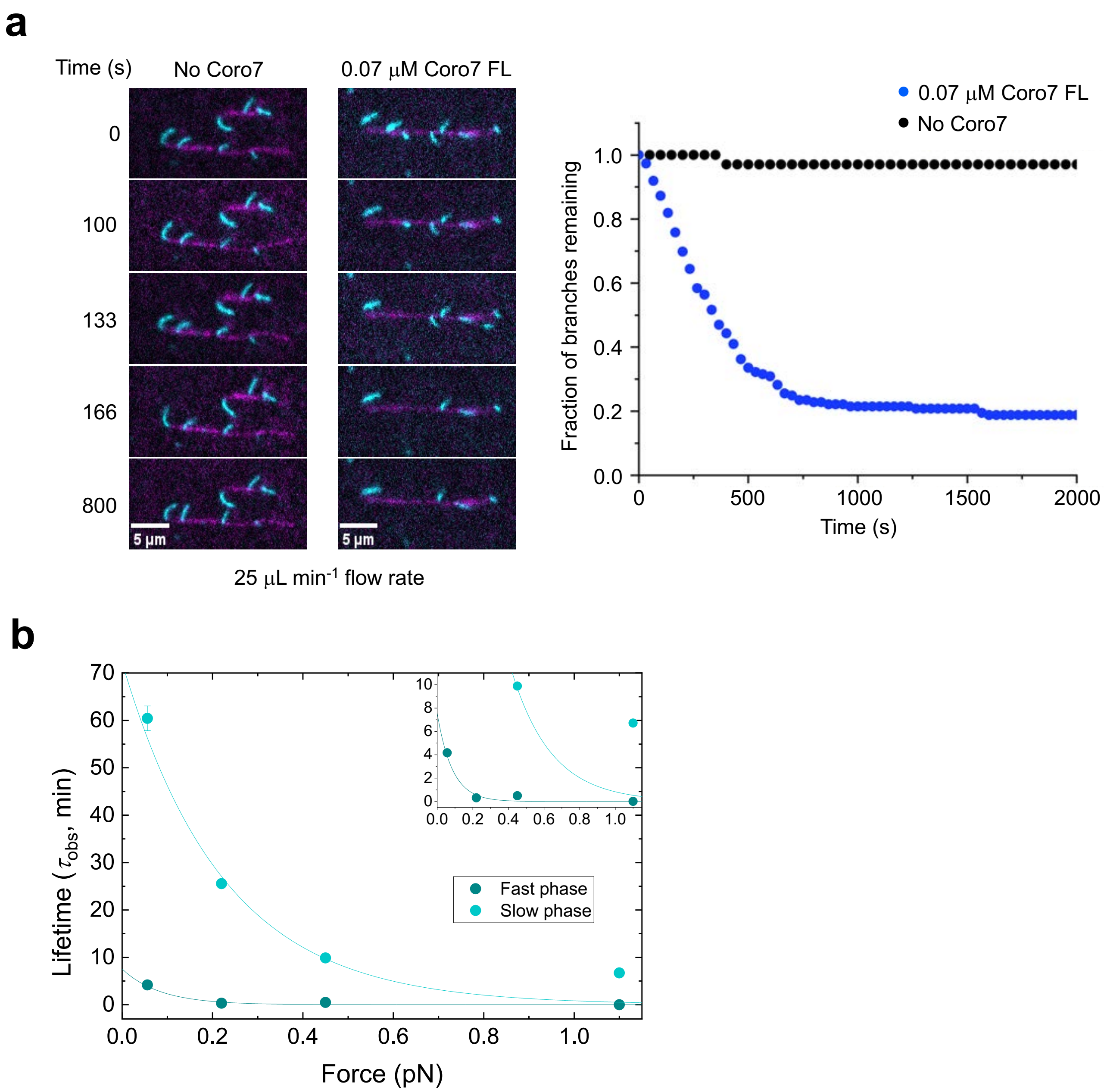

Supplementary Fig. 5 | Force-dependence of branch lifetimes with and without Coro7 FL. a Time-lapse TIRF microscopy images of debranching events in the absence (left) or presence (right) of Coro7 FL. Flow rate is 25 µL min^{-1}, corresponding to an average force of ~0.05 pN on a ~2 µm-long branch. Quantification (right) shows that most branches remain after 13 minutes without Coro7 FL, whereas a substantial fraction dissociates in the presence of 0.07 µM Coro7 FL. **b** Force dependence of Arp2/3 complex branch lifetimes. Data points correspond to the observed lifetimes (τ_{obs}) of the fast and slow debranching phases over a range of applied forces in the presence of 0.07 µM Coro7 (~65% Coro7 occupancy). Solid lines through the data represent best fits to single exponentials of the form of $\tau_{obs} = \tau_0 \exp(-Fd/k_B T)$, where τ_0 is the branch lifetime in the absence of applied force (F), d is the characteristic distance to the transition state – a force sensitivity parameter for branch interface rupture [4], k_B is the Boltzmann constant, and T is the absolute temperature. The inset highlights lifetimes in the 0–10 min range. In the absence of Coro7 (slow phase), branches have an intrinsic lifetime of 72.2 ± 7.6 min in the absence of force and a "half-force" ($F_{1/2}$) of 0.15 ± 0.01 pN. In the presence of Coro7 (fast phase), branches have an intrinsic lifetime of 7.6 ± 6.4 min in the absence of force and a "half force" ($F_{1/2}$) of 0.06 ± 0.03 pN, indicating that Coro7 dramatically increases branch sensitivity to force-induced rupture.

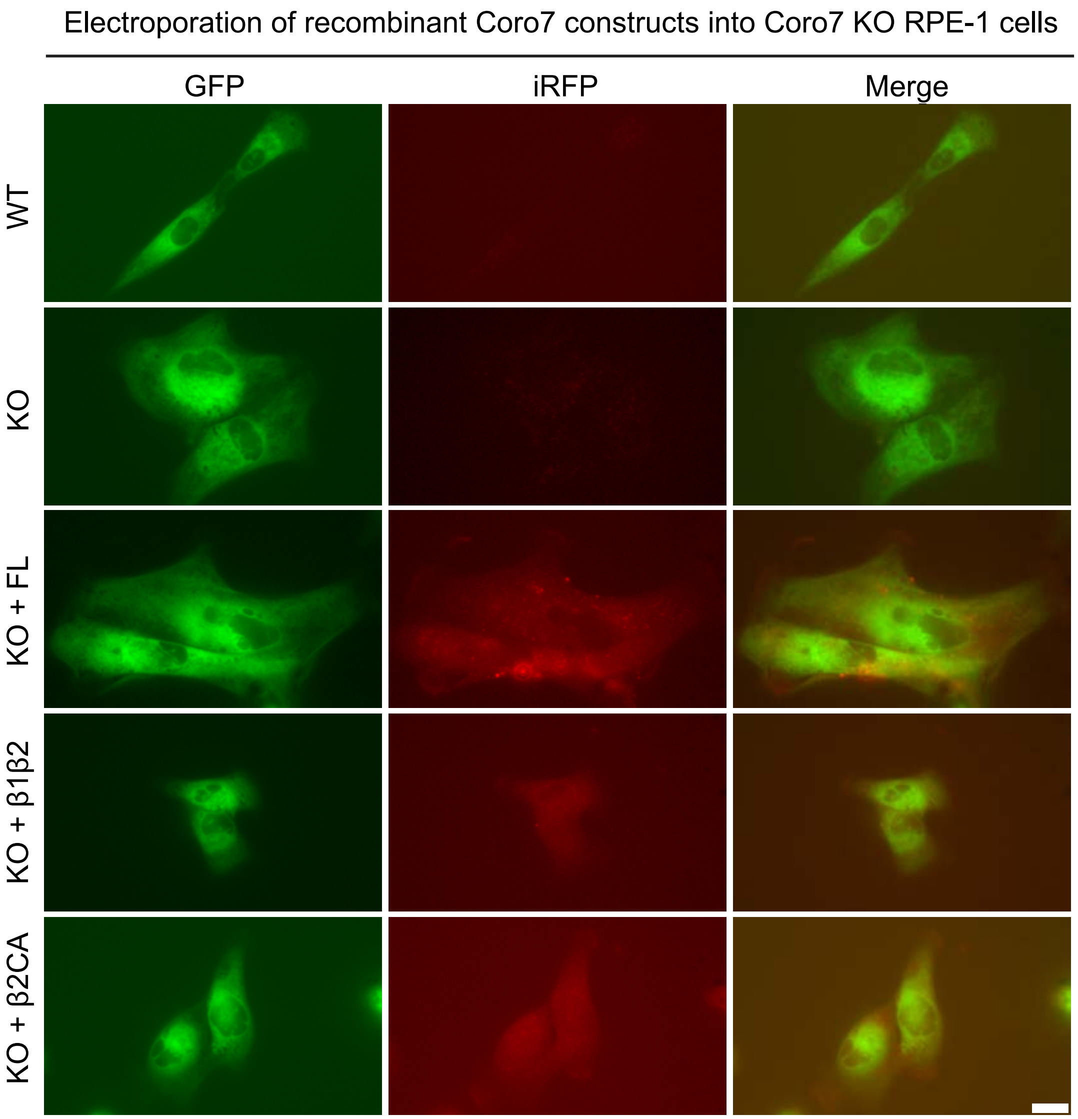

Supplementary Fig. 6 | Electroporation of purified Coro7 proteins into Coro7 KO cells. Electroporation of Flag-iRFP-tagged Coro7 FL, β1β2, and β2CA results in approximately uniform levels of exogenous protein in RPE-1 cells (red). The GFP signal (green) indicates the ER localization of GFP-GPI in the absence of biotin addition to the culture medium. Scale bar: 20 µm.

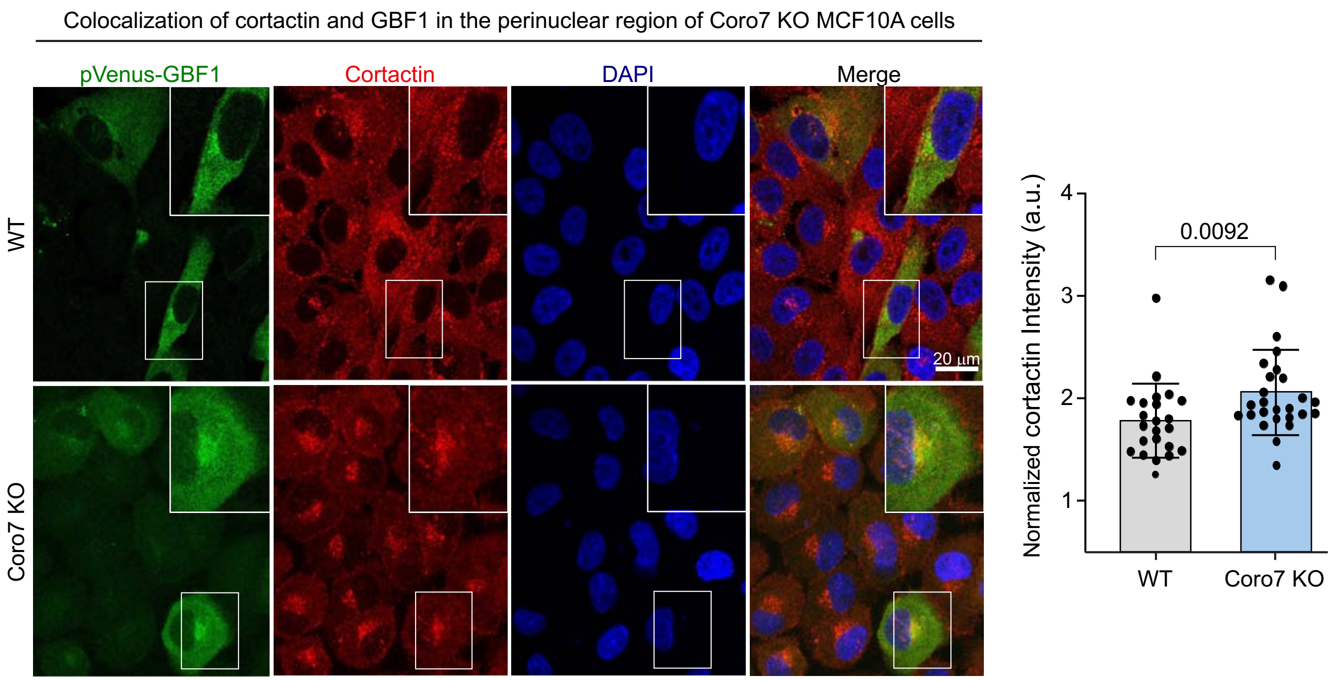

Supplementary Fig. 7 | Loss of Coro7 increases perinuclear branched actin networks that colocalize with GBF1 at the ERGIC. a Parental MCF10A cells or Coro7 KO derivatives were transiently transfected with pVenus-GBF1 and stained 24 hours later with antibodies against cortactin (a branched actin network marker) and DAPI (a nuclear marker). Scale bar: 20 µm. **b** Quantification of mean cortactin intensity in the perinuclear region, defined by pVenus-GBF1 masking, and normalized to the total cortactin intensity per cell in MCF10A parental and Coro7 KO cells. In Coro7 KO cells, normalized cortactin intensity increased by an average of 14% relative to parental MCF10A cells (WT: 1.78 ± 0.36, n = 23 cells; KO: 2.05 ± 0.41, n = 26 cells). The graph shows one representative experiment of three independent experiments. Data are presented as mean ± SD. Statistical significance was calculated using a two-tailed Mann-Whitney U test ($p = 0.0092$). Scale bar: 20 µm. Source data and statistical analysis are provided in the Source Data file.

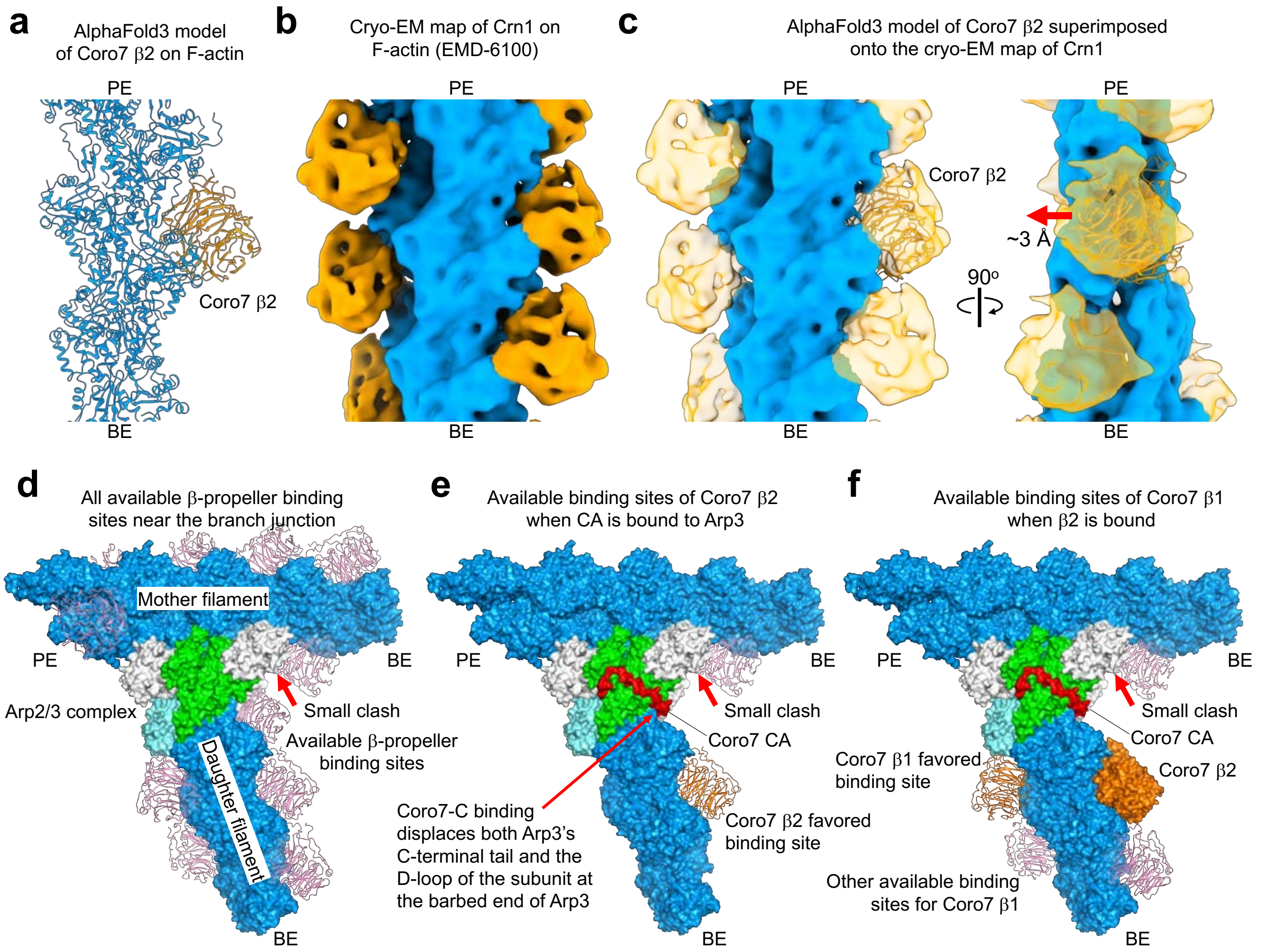

Supplementary Fig. 8 | Evidence supporting the proposed Coro7 debranching model. **a** Model of Coro7 β2 (orange) on F-actin (blue) obtained with AlphaFold3 [5] when run together with seven actin subunits. A nearly identical prediction is obtained for Coro7 β1 (not shown). PE and BE stand for pointed and barbed ends, respectively. **b** Cryo-EM map (8.6-Å resolution) of yeast Crn1 (orange) in complex with F-actin [6]. **c** AlphaFold3 model of Coro7 β2 superimposed onto the cryo-EM map of Crn1. A red arrow indicates that the AlphaFold3 model would need to be translated by ~3 Å to better fit the map. Because the cryo-EM map of yeast Crn1 and the AlphaFold3 models of human Coro7 β-propellers largely agree, we have chosen to use the AlphaFold3 models as reference to produce a model of Coro7 at the branch junction. **d** Structure of the cortactin-bound branch junction (PDB: 8P94, cortactin not shown) [7], illustrating all the available β-propeller binding sites (pink) near the junction. **e** Structure of Coro7 CA fitted onto the branch junction structure, using Arp3 subdomains 3 and 4 as reference for fitting. Note that the binding of Coro7-C displaces both the C-terminal tail of Arp3 (Fig. 3g) and the D-loop of the subunit at the barbed end of Arp3, which we propose serves as the initial trigger for branch disassembly. Due to the short length of the β2-CA linker, once CA is positioned, β2 can potentially reach only two binding sites. One site, on the mother filament, was ruled out due to a small clash with Arp2/3 complex (red arrow). The accepted, most likely site of β2 (orange) is at the interface between the first two actin subunits at the barbed end of Arp3. **f** The β1-β2 linker is sufficiently long that, once β2 is positioned (surface representation, orange), β1 can potentially reach multiple sites on the daughter filament and one site on the mother filament (pink). The site on the mother filament is again excluded due to a small clash with Arp2/3 complex (red arrow). Of the remaining sites on the daughter filament, we favor the one next to Arp2 (ribbon representation, orange), as it would be consistent with greater specificity of Coro7 for the branch junction.

Supplementary Table 1 | Primers and antibodies used in this study

Primers

Coro7 construct	Vector	Forward	Reverse
FL	pMAL	5’ ttcgcggccgcatgaaccgcttcagggtgtcc	5’ ttcgaattcctagtcccactcgtcctcgtcc
β1β2	pMAL	5’ ttcgcggccgcatgaaccgcttcagggtgtcc	5’ tccgaattcctaagacttttcttccaggtactgcgc
β2CA	pMAL	5’ ttcgctagctccaagttccgccatgc	5’ ttcgaattcctagtcccactcgtcctcgtcc
CA	pMAL	5’ ttcgcggccgctctgaccagcaaaagaagg	5’ ttcgaattcctagtcccactcgtcctcgtcc
MBP-FL	pJC7	5’ ttcgtcgacgccaccatgaaaatccaccatcaccaccacc	5’ ttcgaattcctagtcccactcgtcctcg
MBP-β1β2	pJC7	5’ ttcgtcgacgccaccatgaaaatccaccatcaccaccacc	5’ tccgaattcctaagacttttcttccaggtactgcgc
MBP-β2CA	pJC7	5’ ttcgtcgacgccaccatgaaaatccaccatcaccaccacc	5’ ttcgaattcctagtcccactcgtcctcg

Antibodies

Antigen	Dilution	Description	Source (catalog number)
Primary antibodies			
Arp3	1 : 5000	Mouse monoclonal anti-Arp3	Santa Cruz Biotechnology (sc-48344)
Coro7	1 : 500	Rabbit polyclonal anti-Coro7	Abcam (ab117446)
Flag	1 : 250	Mouse monoclonal anti-FLAG BioM2-Biotin	Sigma-Aldrich (F9291)
α-tubulin	1 : 2000	Mouse monoclonal anti-α-tubulin	Sigma-Aldrich (T9026)
Cortactin	1 : 200	Mouse monoclonal anti-cortactin	Sigma-Aldrich (05-180-I)
Secondary antibodies			
Mouse IgG	1 : 10000	Anti-mouse HRP-linked antibody	Cell Signaling Technology (7074S)
Rabbit IgG	1 : 5000	Anti-rabbit HRP-linked antibody	Sigma-Aldrich (12-348)

Supplementary Table 1 | Primers and antibodies used in this study. List of primers (top) and antibodies (bottom) used in this study, including their commercial sources and dilutions used.

Supplementary References

1. Naydenova K, Russo CJ. Measuring the effects of particle orientation to improve the efficiency of electron cryomicroscopy. *Nat Commun* **8**, 629 (2017).

2. Tan YZ*, et al.* Addressing preferred specimen orientation in single-particle cryo-EM through tilting. *Nat Methods* **14**, 793-796 (2017).

3. van Zundert GCP, Moriarty NW, Sobolev OV, Adams PD, Borrelli KW. Macromolecular refinement of X-ray and cryoelectron microscopy structures with Phenix/OPLS3e for improved structure and ligand quality. *Structure* **29**, 913-921 e914 (2021).

4. Pandit NG*, et al.* Force and phosphate release from Arp2/3 complex promote dissociation of actin filament branches. *Proc Natl Acad Sci U S A* **117**, 13519-13528 (2020).

5. Abramson J*, et al.* Accurate structure prediction of biomolecular interactions with AlphaFold 3. *Nature* **630**, 493-500 (2024).

6. Ge P, Durer ZA, Kudryashov D, Zhou ZH, Reisler E. Cryo-EM reveals different coronin binding modes for ADP- and ADP-BeFx actin filaments. *Nat Struct Mol Biol* **21**, 1075-1081 (2014).

7. Liu T, Cao L, Mladenov M, Jegou A, Way M, Moores CA. Cortactin stabilizes actin branches by bridging activated Arp2/3 to its nucleated actin filament. *Nat Struct Mol Biol* **31**, 801-809 (2024).
