# Supplementary material for: Mechanism of Arp2/3 complex branch disassembly by human Coro7
Source: Nat Commun. 2025 Nov 6;16:9809. doi: 10.1038/s41467-025-64787-z (PMC12592476; doi:10.1038/s41467-025-64787-z)
Supplement: Supplementary file 2 — Description of Additional Supplementary Files [file 41467_2025_64787_MOESM2_ESM.pdf]

Title: Supplementary Video 1 – Structure of Coro7 CA bound to Arp2/3 complex.
Description: The movie shows a full rotation of the structure, followed by close-up views of specific interactions involving Coro7-C and Coro7-A. Arp2/3 complex subunits are labeled and shown in different colors, and Coro7 colored red.

Title: Supplementary Video 2 – Arp2/3 complex branch dissociation without Coro7.
Description: Movie of timelapse fluorescence micrographs, captured at 6-second intervals, showing Arp2/3 complex branch dissociation events in the absence of Coro7. Branches are aligned with the direction of the buffer flow. At 522 s, the flow rate increases from 25 to 200 µL min^{-1}, corresponding to an increase in the force applied to a ~2 µm-long branch from ~0.05 to ~0.45 pN. Dissociation events are observed at 588 s, 1115 s, and 1520 s.

Title: Supplementary Video 3 – Arp2/3 complex branch dissociation with Coro7 FL.
Description: Movie of timelapse fluorescence micrographs, captured at 33-second intervals, showing Arp2/3 complex branch dissociation events in the presence of Coro7 FL. Branches are aligned with the direction of the buffer flow. At 300 s, the flow rate increases from 25 to 200 µL min^{-1}, corresponding to an increase in the force applied to a ~2 µm-long branch from ~0.05 to ~0.45 pN. Dissociation events are observed at 330 s and 363 s.

Title: Supplementary Video 4 – Arp2/3 complex branch dissociation with Coro7 β2CA.
Description: Movie of timelapse fluorescence micrographs, captured at 25-second intervals, showing Arp2/3 complex branch dissociation events in the presence of Coro7 β_2CA. Branches are aligned with the direction of the buffer flow. At 270 s, the flow rate increases from 25 to 200 µL min^{-1}, corresponding to an increase in the force applied to a ~2 µm-long branch from ~0.05 to ~0.45 pN. Dissociation events are observed at 275 s, 300 s, 425 s, and 550 s.

Title: Supplementary Video 5 – Arp2/3 complex branch dissociation with Coro7 β1β2.
Description: Movie of timelapse fluorescence micrographs, captured at 20-second intervals, showing Arp2/3 complex branch dissociation events in the presence of Coro7 $\beta_1\beta_2$. Branches are aligned with the direction of the buffer flow. At 290 s, the flow rate increases from 25 to 200 µL min^{-1}, corresponding to an increase in the force applied to a ~2 µm-long branch from ~0.05 to ~0.45 pN. Dissociation events are observed at 300 s, 620 s, 1000 s, and 1460 s.

Title: Supplementary Video 6 – Arp2/3 complex branch dissociation with Coro7 CA.
Description: Movie of timelapse fluorescence micrographs, captured at 25-second intervals, showing Arp2/3 complex branch dissociation events in the presence of Coro7 CA. Branches are aligned with the direction of the buffer flow. At 125 s, the flow rate increases from 25 to 200 µL min^{-1}, corresponding to an increase in the force applied to a ~2 µm-long branch from ~0.05 to ~0.45 pN. Dissociation events are observed at 150 s and 975 s.

Title: Supplementary Video 7 – Trafficking of GFP-GPI in RPE-1 cells.
Description: Trafficking of the GFP-GPI signal in RPE-1 parental cells (WT), Coro7 KO cells, and KO cells electroporated with purified Flag-iRFP-tagged Coro7 FL, β1β2, and β2CA proteins. Scale bar: 20 μm.

Title: Supplementary Video 8 – Proposed model of branch disassembly by Coro7.
Description: The video begins with a surface representation of the structure of the branch junction with bound cortactin (PDB: 8P94, cortactin not shown), alongside a ribbon representation of an AlphaFold3 model of human Coro7. Actin subunits of the mother and branch filaments are colored blue. Arp2/3 complex subunits are gray, except for Arp2 (cyan) and Arp3 (green). The β-propellers of Coro7 are colored orange, and the linkers and CA region are colored red. Starting from the AlphaFold3 model, Coro7 must undergo a conformational change that exposes its actin- and Arp2/3 complex-binding surfaces. Coro-A (A region of CA) and β2 might bind first**.** Coro-A interacts with Arp3, adopting the conformation observed in the cryo-EM structure (Fig. 3a). Given the length of the β2-CA linker, β2 can only bind to two positions—one on the mother filament and one on the daughter filament (Supplementary Fig. 8e). After ruling out the position on **the** mother filament due to a small steric clash, β2 is proposed to bind at the interface between the first two actin subunits at the barbed end of Arp3. The specific contacts of β2 (and β1) with F-actin are based on AlphaFold3 predictions (ran together with seven actin subunits), and are further consistent with the cryo-EM map of yeast Crn1 in complex with F-actin (Supplementary Fig. 8b). The β1-β2 linker is long, in principle allowing β1 to bind in multiple locations (Supplementary Fig. 8f) after β2 is positioned. In our model, β1 is positioned at the interface of Arp2 and the actin subunit at its barbed end, on the opposite long-pitch helix relative to β2, which we suggest may enhance Coro7's specificity for the branch junction. Debranching is proposed to be initiated by Coro7-C (C region of CA) inserting into the hydrophobic cleft of Arp3, displacing the D-loop of the actin subunit bound at the barbed end of Arp3. This destabilizes the interface between Arp2/3 complex and the daughter filament. Regardless of whether concurrent destabilization occurs on the Arp2 side, this model predicts that the branch ultimately detaches. Coro7 may remain transiently bound to either the pointed end of the branch or Arp2/3 complex. Once the branch detaches, Arp2/3 complex dissociates from the mother filament and reverts to its inactive conformation.
