# Supplementary material for: Mechanism of Arp2/3 complex branch disassembly by human Coro7
Source: Nat Commun. 2025 Nov 6;16:9809. doi: 10.1038/s41467-025-64787-z (PMC12592476; doi:10.1038/s41467-025-64787-z)
Supplement: Supplementary file 11 — Reporting Summary [file 41467_2025_64787_MOESM11_ESM.pdf]

nature portfolio

Corresponding author(s): Roberto Dominguez

Last updated by author(s): 2025/09/04

Reporting Summary

Nature Portfolio wishes to improve the reproducibility of the work that we publish. This form provides structure for consistency and transparency in reporting. For further information on Nature Portfolio policies, see our Editorial Policies and the Editorial Policy Checklist.

Statistics

For all statistical analyses, confirm that the following items are present in the figure legend, table legend, main text, or Methods section.

n/a	Confirmed	
☐	☒	The exact sample size (*n*) for each experimental group/condition, given as a discrete number and unit of measurement
☐	☒	A statement on whether measurements were taken from distinct samples or whether the same sample was measured repeatedly
☐	☒	The statistical test(s) used AND whether they are one- or two-sided<br>*Only common tests should be described solely by name; describe more complex techniques in the Methods section.*
☒	☐	A description of all covariates tested
☒	☐	A description of any assumptions or corrections, such as tests of normality and adjustment for multiple comparisons
☐	☒	A full description of the statistical parameters including central tendency (e.g. means) or other basic estimates (e.g. regression coefficient) AND variation (e.g. standard deviation) or associated estimates of uncertainty (e.g. confidence intervals)
☐	☒	For null hypothesis testing, the test statistic (e.g. *F*, *t*, *r*) with confidence intervals, effect sizes, degrees of freedom and *P* value noted<br>*Give P values as exact values whenever suitable.*
☒	☐	For Bayesian analysis, information on the choice of priors and Markov chain Monte Carlo settings
☒	☐	For hierarchical and complex designs, identification of the appropriate level for tests and full reporting of outcomes
☒	☐	Estimates of effect sizes (e.g. Cohen's *d*, Pearson's *r*), indicating how they were calculated

Our web collection on statistics for biologists contains articles on many of the points above.

Software and code

Policy information about availability of computer code

Data collection	Cryo-EM data was collected using EPU software (v.3.0) on a 300 kV Titan Krios electron microscope equipped with a Gatan K3 direct electron detector and energy quantum filter (Thermo Fisher Scientific).<br>Protein gels and Westerns were imaged using GENESys V1.5.6.0 (Genesys Limited).<br>Isothermal titration calorimetry experiments were performed using a VP-ITC instrument (MicroCal).<br>Pyrene polymerization assays were performed on Cary Eclipse fluorescence spectrophotometer (Varian).<br>Time-lapse imaging was performed at 37 °C in a thermostat-controlled chamber using a Zeiss epifluorescence microscope.
Data analysis	Processing of cryo-EM data was performed in CryoSPARC (v.4.6.0). The orientation distribution of particles in the consensus map was evaluated using cryoEF (v.1.1.0) and 3DFSC (https://3dfsc.salk.edu/). Model building and refinement was performed using Coot (v.0.9.8.3) and Phenix (v.1.20.1). Data from pyrene-actin polymerization, assays, crosslinking assays, cell biology , microfluidics-TIRF, and cosedimentation were plotted and statistical analyses were performed using Prism (v.9.5.1). Structure figures were prepared using ChimeraX (v.1.4) and PyMOL (v.2.5.0). For ITC, Origin (v.7.0) was used to analyze the raw binding isotherms. Sequence alignment was performed in Jalview (v.2.11.4.1). Densitometric quantification of western blots and protein gels was performed using ImageLab (v.6.0.1). PowerPoint (v.16.89) was used to compile and prepare all other figures.

For manuscripts utilizing custom algorithms or software that are central to the research but not yet described in published literature, software must be made available to editors and reviewers. We strongly encourage code deposition in a community repository (e.g. GitHub). See the Nature Portfolio guidelines for submitting code & software for further information.

Data

Policy information about availability of data

All manuscripts must include a data availability statement. This statement should provide the following information, where applicable:

- Accession codes, unique identifiers, or web links for publicly available datasets
- A description of any restrictions on data availability
- For clinical datasets or third party data, please ensure that the statement adheres to our policy

Data availability
Cryo-EM maps, models, and micrographs were deposited with the EMDB, PDB, and EMPIAR repositories. Accession codes are as follows: EMD- 47408 [https://www.ebi.ac.uk/emdb/EMD-47408] (consensus map); EMD-47746 [https://www.ebi.ac.uk/emdb/EMD-47746], EMD-47769 [https://www.ebi.ac.uk/emdb/EMD-47769], EMD-47770 [https://www.ebi.ac.uk/emdb/EMD-47770], EMD-47772 [https://www.ebi.ac.uk/emdb/EMD-47772], EMD-47795 [https://www.ebi.ac.uk/emdb/EMD-47795] and EMD-47797 [https://www.ebi.ac.uk/emdb/EMD-47797] (locally refined and focused maps); EMD-47836 [https://www.ebi.ac.uk/emdb/EMD-47836] (composite map); PDB 9EAM [https://www.rcsb.org/structure/unreleased/9EAM] (coordinates); and EMPIAR-12575 [https://www.ebi.ac.uk/empiar/EMPIAR-12575/] (micrographs). Source data are provided with this paper.

Research involving human participants, their data, or biological material

Policy information about studies with human participants or human data. See also policy information about sex, gender (identity/presentation), and sexual orientation and race, ethnicity and racism.

Reporting on sex and gender	Research not involving human participants or human data
Reporting on race, ethnicity, or other socially relevant groupings	Research not involving human participants or human data
Population characteristics	Research not involving human participants or human data
Recruitment	Research not involving human participants or human data
Ethics oversight	Research not involving human participants or human data

Note that full information on the approval of the study protocol must also be provided in the manuscript.

Field-specific reporting

Please select the one below that is the best fit for your research. If you are not sure, read the appropriate sections before making your selection.

[x] Life sciences [ ] Behavioural & social sciences [ ] Ecological, evolutionary & environmental sciences

For a reference copy of the document with all sections, see nature.com/documents/nr-reporting-summary-flat.pdf

Life sciences study design

All studies must disclose on these points even when the disclosure is negative.

Sample size	Sample sizes for pyrene-actin polymerization assays of N ≥ 3 was chosen for each condition based on previous experiences with this specific type of experiment and commonly used sample size in the field (Madasu et al 2015, Drazic et al 2018, Zimmet et al 2020, Fregoso et al 2022, Fregoso et al 2023). For crosslinking and cosedimentation assays a smaple size was N ≥ 3 for each condition.<br>For cell biology experiments, between 28 and 62 cells were analyzed for each experimental condition.
Data exclusions	No data were excluded from analysis, unless experiments failed for obvious reasons
Replication	All biochemical and cell biological experiments in this study have been reproduced using the same experimental set-ups (i.e., proteins, buffers, cell extracts, etc.) with similar results. All Western blots and protein gels are shown. The numbers of independent replications are indicated in the Figure Legends.
Randomization	Randomization was not necessary.
Blinding	CryoSPARC (v4.6.4) is a generally applied and accepted software for cryo-EM data processing that inherently processes data in an unbiased manner from the investigator.

Reporting for specific materials, systems and methods

We require information from authors about some types of materials, experimental systems and methods used in many studies. Here, indicate whether each material, system or method listed is relevant to your study. If you are not sure if a list item applies to your research, read the appropriate section before selecting a response.

Materials & experimental systems

n/a	Involved in the study
☐	☒ Antibodies
☐	☒ Eukaryotic cell lines
☒	☐ Palaeontology and archaeology
☒	☐ Animals and other organisms
☒	☐ Clinical data
☒	☐ Dual use research of concern
☒	☐ Plants

Methods

n/a	Involved in the study
☒	☐ ChIP-seq
☒	☐ Flow cytometry
☒	☐ MRI-based neuroimaging

Antibodies

Antibodies used	anti-Arp3 (sc-48344), anti-Coro7 (ab117446), anti-FLAG BioM2-Biotin (Sigma-Aldrich F9291), anti-α-tubulin (Sigma-Aldrich T9026), anti-cortactin (Sigma-Aldrich 05-180-I),anti-mouse HRP (Cell Signaling Technology 7074S), anti-rabbit HRP (Sigma-Aldrich 12-348).
Validation	All antibody are commercial, validated by manufacturer.

Eukaryotic cell lines

Policy information about cell lines and Sex and Gender in Research

Cell line source(s)	Expi293F cells were obtained from ThermoFisher Scientific. HEK293T were obtained from Sigma-Aldrich. hTERT-immortalized RPE-1 human retinal pigment epithelial cells, stably expressing the Str-KDEL_SBP-EGFPGPI plasmid for imaging GFP-GPI trafficking in the RUSH system, were provided by Gaëlle Boncompain and Franck Perez (Institut Curie, Paris), MCF10A cells were a gift from T. Dubois (Institut Curie, Paris).
Authentication	Expi293F cells and HEK293T are authenticated by manufacturer.
Mycoplasma contamination	Expi293F cells and HEK293T cells were confirmed negative for mycoplasma contamination. hTERT-immortalized RPE-1 cells and MCF10A cells were not tested.
Commonly misidentified lines (See ICLAC register)	none

Plants

Seed stocks	not applicable
Novel plant genotypes	not applicable
Authentication	not applicable
